# Ontario Veterinary Association

**Published:** 1883-04

**Authors:** 


### ONTARIO VETERINARY ASSOCIATION.

The Annual Meeting of the Ontario Veterinary Association was held in the Ontario Veterinary College, Toronto, on Thursday, December 21st, 1882.

Members attended from all parts of the Province. Also some from the United States.

The President, Mr. Elliot, in his opening address referred to the advaucement of the profession in Canada, and citing as proof of the confidence of the public in its members that not a dollar of the funds of the Association had to be expended in defending members in law courts. He expressed the opinion that a beneficent society in connection with the Association would be appreciated by its members, and closed his remarks with a well merited eulogium of the Ontario Veterinary College.

The minutes of the last meeting were then read and confirmed, and the Secretary and Treasurer's report read and adopted, showing the finances of the Association to be in a healthy state.

Dr. Duncan moved, seconded by Mr. Wilson, supplemented by some very complimentary remarks by Professor Smith, that in view of the great services rendered to the veterinary profession by George Fleming, Esq., F.R.C.V.S, through his valuable contributions to veterinary literature through his exertions in the passage of the Veterinary Act of 1881 and in other ways, therefore be it

*Resolved*, That the Ontario Veterinary Association in behalf of Colonial practitioners records its high appreciation of the labors of Mr. Fleming, and requests its Treasurer to forward the sum of twenty-five dollars as a contribution towards the testimonial about to be presented to that gentleman in acknowledgment of these services.

The resolution was carried unanimously.

Mr. Cowan moved, seconded by Mr. Coleman, that the Association having heard with pleasure of the honor that had been conferred on Professor Smith, Principal of the Ontario Veterinary College, by electing him an honorary Associate of the Royal College of Veterinary Surgeons, desired to express its appreciation of the honor conferred on the respected Professor of the Ontario College, and through him on the veterinary profession on this continent. Carried.

Dr. Duncan in addressing the meeting expressed the hope that increased interest would be taken in the meetings, and that members should regularly read and discuss papers at each meeting.

Messrs. Rogers, Cowan and Sweetapple agreed to read papers at the next meeting.

Mr. Hinman gave a very interesting account of a peculiar case in his practice.

Attention was called to a person advertising illegally as a veterinary surgeon, and the secretary was instructed to notify him to discontinue so doing.

Some matters relating to the tariff of fees were then discussed.

Several new members were duly elected.

The election of office-bearers for the ensuing year then took place with the following results: Mr. C. Elliot, re-elected President; Mr. Coleman, First Vice-President; Mr. O'Neil, Second Vice-President; Mr. Sweetapple, Secretary; Mr. Cowan, Treasurer; Messrs. Hamilton and Hinman, Auditors; Messrs. Hinman, Sanderson, Hamilton, Cæsar, Wilson, Logan, Steele and Grange, Directors; Honorary Director, Professor Smith.

Moved by Mr. Wilson, seconded by Mr. O'Neil, that the sum of twenty-five dollars be appropriated for a medal to be competed for by the students of the Ontario Veterinary College at the Spring examination. Carried.

The meeting then adjourned to meet again in the Spring.

